# Increased Notch Signaling Enhances Radioresistance of Malignant Stromal Cells Induced by Glioma Stem/ Progenitor Cells

**DOI:** 10.1371/journal.pone.0142594

**Published:** 2015-11-23

**Authors:** Yuntian Shen, Hua Chen, Jinshi Zhang, Yanming Chen, Mengyao Wang, Jiawei Ma, Lei Hong, Ning Liu, Qiuhong Fan, Xueguan Lu, Ye Tian, Aidong Wang, Jun Dong, Qing Lan, Qiang Huang

**Affiliations:** 1 Department of Neurosurgery, The Second Affiliated Hospital of Soochow University, Suzhou, China; 2 Department of Radiotherapy & Oncology, The Second Affiliated Hospital of Soochow University; Institute of Radiotherapy & Oncology, Soochow University; Suzhou Key Laboratory for Radiation Oncology, Suzhou, China; 3 Laboratory Center, The Second Affiliated Hospital of Soochow University, Suzhou, China; 4 Department of Neurosurgery, Nanjing First Hospital, Nanjing Medical University, Nanjing, China; 5 Department of Neurosurgery, The First Affiliated Hospital of Nanjing Medical University, Nanjing, China; Swedish Neuroscience Institute, UNITED STATES

## Abstract

**Background:**

Host malignant stromal cells induced by glioma stem/progenitor cells were revealed to be more radiation-resistant than the glioma stem/progenitor cells themselves after malignant transformation in nude mice. However, the mechanism underlying this phenomenon remains unclear.

**Methods:**

Malignant stromal cells induced by glioma stem/progenitor cell 2 (GSC-induced host brain tumor cells, ihBTC2) were isolated and identified from the double color-coded orthotopic glioma nude mouse model. The survival fraction at 2 Gy (SF2) was used to evaluate the radiation resistance of ihBTC2, the human glioma stem/progenitor cell line SU3 and its radiation-resistant sub-strain SU3-5R and the rat C6 glioma cell line. The mRNA of Notch 1 and Hes1 from ihBTC2 cells were detected using qPCR before and after 4 Gy radiation. The expression of the Notch 1, pAkt and Bcl-2 proteins were investigated by Western blot. To confirm the role of the Notch pathway in the radiation resistance of ihBTC2, Notch signaling blocker gamma secretase inhibitors (GSIs) were used.

**Results:**

The ihBTC2 cells had malignant phenotypes, such as infinite proliferation, hyperpentaploid karyotype, tumorigenesis in nude mice and expression of protein markers of oligodendroglia cells. The SF2 of ihBTC2 cells was significantly higher than that of any other cell line (*P*<0.05, n = 3). The expression of Notch 1 and Hes1 mRNAs from ihBTC2 cells was significantly increased after radiation. Moreover, the Notch 1, pAkt and Bcl-2 proteins were significantly increased after radiation (P<0.05, n = 3). Inhibition of Notch signaling markedly enhanced the radiosensitivity of ihBTC2 cells.

**Conclusions:**

In an orthotopic glioma model, the malignant transformation of host stromal cells was induced by glioma stem/progenitor cells. IhBTC2 cells are more radiation-resistant than the glioma stem/progenitor cells, which may be mediated by activation of the Notch signaling pathway.

## Introduction

Currently, drug resistance, the spread of tumors and the role of normal tissue are three mysteries for tumor researchers [1]. Normal tissues originating from host cells are no longer normal tissues or cells after they become part of the micro-environment of tumors [2]. For example, tumor-associated macrophages are associated cells of the tumor with qualities that are altered to promote tumor growth [3]. When orthotopically transplanting the human glioma stem/progenitor cells line SU3 transfected with the red fluorescence protein gene (SU3-RFP) into nude mice expressing enhanced green fluorescent protein gene (EGFP), we established two malignant host cells lines from transplanted tumors. Of these, the second cell line was identified as an oligodendrocyte-like cell line, using immunocytochemical staining. Because they are malignant host cells induced by human glioma stem/progenitor cells, they are named GSC-induced host brain tumor cells (ihBTC1 and ihBTC2). This cell line is confirmed to be cancer with immortalized characteristics *in vitro* and tumorigenicity *in vivo* and greater radiation-resistance than the glioma stem/progenitor cell SU3 and its radiation sub-strain SU3-5R. This new discovery may be helpful in solving the puzzle of the “role of normal tissues” in tumorigenesis, tumor progression and resistance to treatment, particularly radiotherapy.

## Materials and Methods

### Materials

The human glioma stem/progenitor cell line SU3 and nude mice expressing EGFP (NC-CB57/6J-EGFP) were prepared by our laboratory [4, 5]. The remaining reagents were purchased from companies as follows: rat C6 glioma cell line (Shanghai Institutes for Biological Sciences), RFP transgenic kit (Genechem, Shanghai), γ-secretase inhibitors DAPT (GSI-IX) (Selleck), Dulbecco's Modified Eagle's Medium (Gibco), fetal bovine serum (Hyclone), Caspase—Glo 3/7 assay kit (Promega), Trizol solution (Invitrogen), reverse transcription kit (Fermentas), ECL chemiluminescence reagent, trypan blue, GAPDH antibody (Biyuntian, Shanghai), Notch-1, NICD, Bcl-2 and pAKT antibodies (Cell Signal), 2', 3'-cyclic nucleotide 3' phosphodiesterase (CNP) monoclonal antibody (Abcam), qPCR apparatus and SYBR Green qPCR Mix (BioRad), apoptosis kit (BD), flow cytometry instrument (Beckman), inverted fluorescence microscope (Carl Zeiss), linear accelerator (Siemens Primus), and in vivo fluorescence imaging system (Maestro EX, CRi).

### Methods

#### Isolation and identification of ihBTCs

RFP was transfected into SU3 cells according to the manufacturer’s protocol provided with the transfection kit. Nude mice were anesthetized by intraperitoneal injection of chloral hydrate because of the short half-life. SU3-expressing RFP cells (SU3-RFP; 1X10^5^, 25 μl) were transplanted into the right caudate nucleus of nude mice expressing EGFP at six weeks of age [6]. The health of the nude mice was monitored each day after transplantation. An in vivo fluorescence imaging system was used to observe the transplanted tumor size. When the widest tumor diameter was greater than 5 mm, the tumor-bearing nude mice were sacrificed under anesthesia. Tumor specimens were cut into small pieces, digested and filtered. The resulting single cell suspensions were cultured in DMEM containing 10% fetal bovine serum. EGFP^+^ colonies were selected and expanded. Then, we used flow cytometry and a capillary pipet to isolate single EGFP^+^ host stromal cell. Ultimately, we established two EGFP^+^ host cells lines with the ability to proliferate indefinitely *in vitro* (ihBTC1 and ihBTC2 cells). The strength of EGFP expression and the cell growth of ihBTCs were observed under an inverted fluorescence microscope. Neural markers of ihBTCs were detected using immunofluorescence staining. The malignant transformation of ihBTC was identified using chromosome karyotype analysis and tumorigenesis experiments in nude mice.

#### Radiation dose survival curve of ihBTC and control cells

SU3 cells in the logarithmic growth phase were irradiated with 4 Gy five times, with a time interval of 7 days. SU3 cells with excellent growth conditions after irradiation were selected and used to establish five radiation-resistant sub-strains named SU3-5. Then, the radiation dose survival curves of ihBTC2, SU3, SU3-5R and C6 cells were constructed. Briefly, the cell suspensions of the different cell lines were adjusted to an appropriate concentration and inoculated into a 6-well plate (N = 3). After complete adherence, the cells were irradiated with either 0, 2, 4, or 6 Gy. Irradiation parameters were 6 MVX, the skin distance was 100 cm and the actual dose rate was 2 Gy/min. Ten days after irradiation, the cell clones in the 6-well plates were washed with phosphate buffered saline (PBS) 2 times, fixed with 4% paraformaldehyde for 30 min, stained with crystal violet and finally washed with PBS. The cell clone (≥ 50 cells) numbers in each well were counted under a microscope. The clone formation rate was then calculated as the number of clones/total number of seeded cells ×100%. The cell survival fraction was the clone formation rate of the experimental group/clone formation rate of the control group (0 Gy). Using the Sigma Plot 9.0 software, the radiation sensitivity parameters survival fraction at 2 Gy (SF2) and cell radiation dose survival curves were obtained using the line-quadratic model.

#### Detection of ihBTC2 radiation resistance associated molecules

First, the expression of the Notch1 mRNA and protein in SU3, SU3-5R and ihBTC2 cells before and after irradiation with 4 Gy were detected using qPCR [7] and western blot [8], respectively. Notch1 downstream target genes were measured using qPCR. The primer sequences are shown as follows: reference gene β-actin-F: 5’-TCCTGTGGCATCCGCGAAACT-3’ and β-actin-R: 5’-GAAGCATTTGCGGTGGACGAT-3’; target genes Notch1-F: 5’-TCTGTGTGGATGAGGGAGATAA-3’ and Notch1-R: 5’-AGCCGCCGAGATAGTCAGT-3’; Jag1-F: 5’-CTGTCAGGTTGAACGGTGTC-3’ and Jag1-R: 5’-CTTCAACCTCAAGGCCAGC-3’; Hes1-F: 5’-CCAGCCAGTGTCAACACGA-3’ and Hes1-R: 5’-AATGCCGGGAGCTATCTTTCT-3’. The Delta-delta Ct method was used to calculate the relative value of the target genes in the different samples.

To further verify the role of the Notch signaling pathway in ihBTC2 radiation resistance, we used two experimental groups. One group was irradiated after administration of Notch pathway blocker gamma-secretase inhibitors (GSI; ihBTC2-radiation combined with GSI group), and another group was only irradiated with 4 Gy (ihBTC2-radiation group). The phosphorylated Akt and Bcl-2 protein expression of the two groups was assessed via western-blot. The MTT assay was used to determine the proliferation curve of the two groups [9]. The sensitivity enhancement ratio (SER) of the GSI was calculated by constructing the cell radiation dose survival curve of the two groups. To detect cell apoptosis, Caspase-Glo 3/7 assay kits, trypan blue staining analysis and flow cytometry were used to measure Caspase 3/7, the dead cell rate and Annexin V staining, respectively.

#### Statistical analysis

The results are expressed as the mean ± standard deviation (SD), and a Student’s t test was used to evaluate statistical significance. A two-tailed Student's t-test was used to compare two independent groups. A P value <0.05 was considered statistically significant. The statistical analyses were performed using the SPSS 13.0 software package.

#### Statement of ethics

Research reported in this manuscript was performed with the approval of the ethics committee of the 2nd affiliated hospital of Soochow University. Animal studies were approved by the Soochow University Animal Care and Use Committee, and they followed internationally recognized guidelines. Animals were euthanized by slow (20%/minute) displacement of chamber air with compressed CO_2_ delivered through a precision flowmeter. The animals were subject to cervical dislocation as a secondary means to ensure death. All efforts were made to minimize suffering. Details of the animal welfare and steps taken to ameliorate suffering were in accordance with the Regulations for the Administration of Affairs Concerning Experimental Animals approved by the State Council of the People’s Republic of China.

## Results

### Cancerous phenotype of ihBTC2

**Fig 1:**
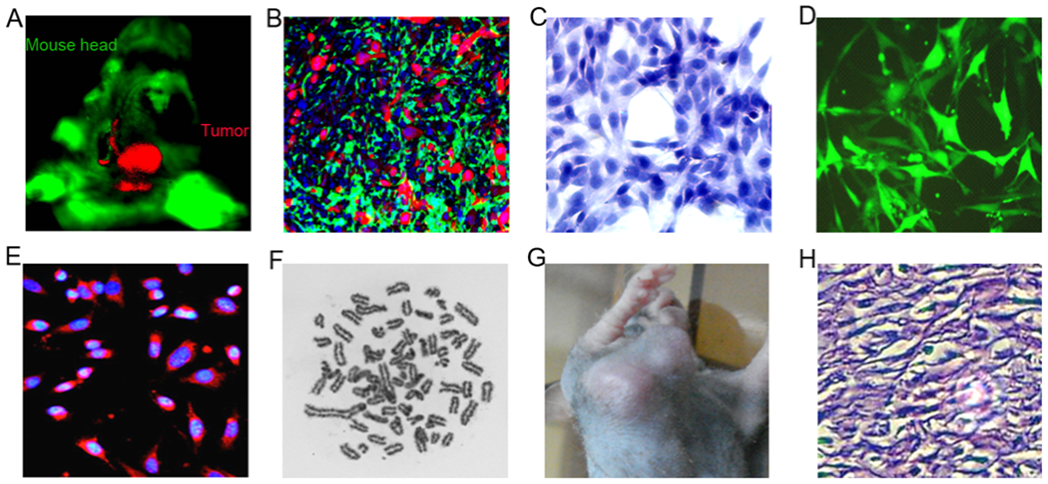
Origin of ihBTCs. A, Under the in vivo fluorescence imaging system, SU3-RFP xenotransplanted tumors in the right caudate nucleus of EGFP nude mice were red. B, Red tumor cells interweave with the green host cells in the xenotransplanted tumor frozen section observed under a fluorescent microscope (scale plate 20 μm). C, HE staining of ihBTC cells. D-E, (scale plate of 50 μm) IhBTC cells express EGFP in in vitro culture. Immunofluorescence reveals that ihBTC2 cells highly express the oligodendrocyte marker CNP (in red and DAPI in blue). F, Karyotype analysis indicates that ihBTC2 cells are hyperpentaploid and all chromosomes are monocentric. G and H, Large tumor formed in non-fluorescent nude mice after subcutaneous injection of ihBTC2 cells. H&E staining of the tumors showed the malignant tumor features of poor differentiation, pleomorphism, hyperchromatic nuclei, loss of polarity and local invasion (200×).

In the double color-coded orthotopic glioma nude mouse model, tumor cells express red fluorescence protein (RFP), and host cells express enhanced green fluorescence protein (EGFP). IhBTC2 cells showed high proliferative activity and lost contact inhibition of growth during the early stage in vitro (less than five passages). The chromosome karyotype analysis indicated that the ihBTC2 cells were hyperpentaploid. IhBTC2 cells expressed the oligodendroglia cell marker CNP. When ihBTC2 cells were implanted subcutaneously into non-fluorescent nude mice, large oligodendroglioma-like lesions formed in all mice (10/10; Fig 1). The features above did not change after multiple rounds of cryopreservation and recovery over 24 months.

### Radiation resistance of ihBTC2 cells

**Fig 2:**
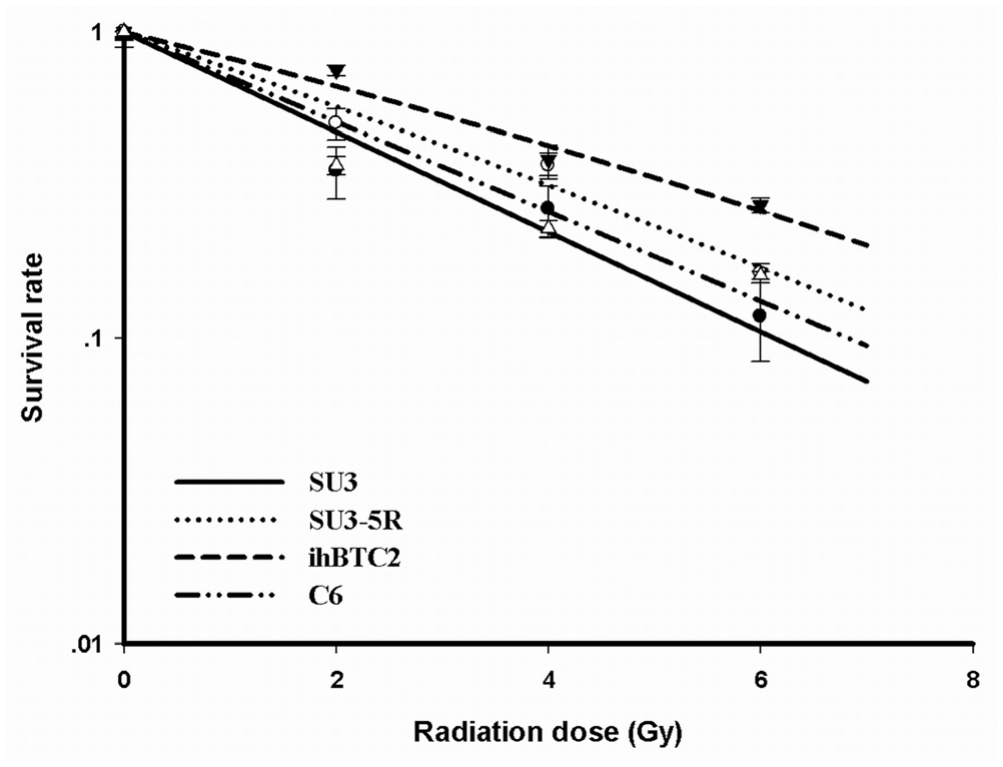
Cell radiation dose-survival curve. The survival rate of the four cell lines decreases as the radiation dose increases in a dose-dependent manner. For radiation resistance, ihBTC2, SU3-5R, C6 and SU3 cells are in descending order.

According to the radiation dose-survival curve, ihBTC2 cells showed obvious radiation resistance. To exclude differences induced by species diversity, the rat C6 glioma cell line was also used. The results indicated that the clone formation rate of SU3, SU3-5R, C6 and ihBTC2 cells without radiation were 1.13±0.12%, 3.27±0.61%, 16.33±0.76% and 8.00±0.50%, respectively. The SF2 values of SU3, SU3-5R, C6 and ihBTC2 cells were 0.35±0.07, 0.50±0.06, 0.51±0.02 and 0.75±0.03, respectively. Statistical analysis showed that ihBTC2 cells were the most radiation resistant cell line of these groups, and SU3-5R had greater radiation resistance than SU3 (P<0.05, N = 3). The difference in radiation resistance became more significant as the radiation dose increased (Fig 2).

### Radiation resistance of ihBTC2 cells is related to Notch pathway activation

**Fig 3:**
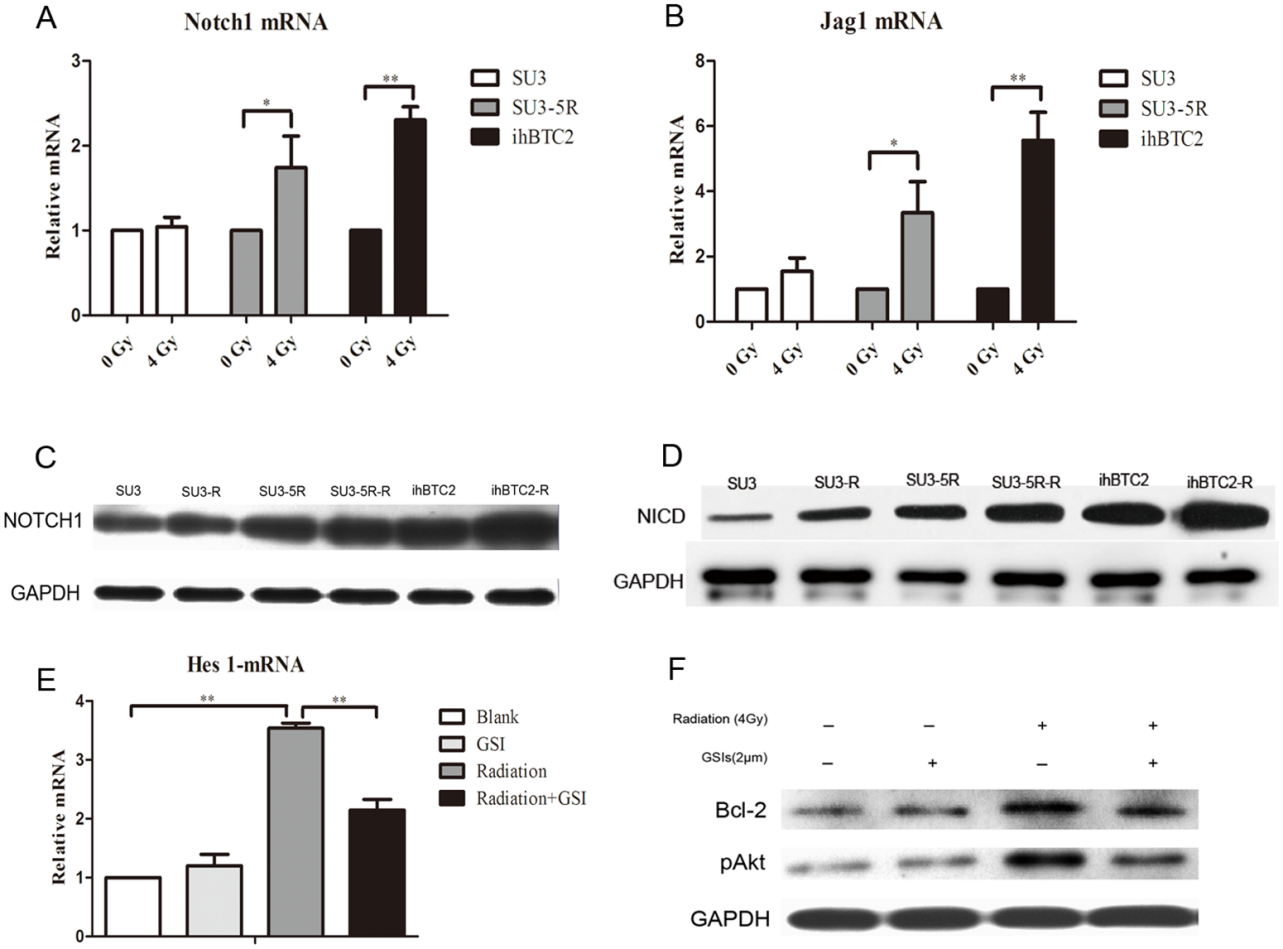
Notch1/Hes1 signaling pathway activation enhances the radiation resistance of ihBTC cells. A-B, Expression of Notch1 and Jag1 mRNA of SU3-5R and ihBTC2 cells increases after radiation (P<0.05). C-D, Notch1 and NICD protein of SU3-5R and ihBTC2 increased after radiation (P<0.05). E, GSI significantly inhibited the up-regulation of Hes1 mRNA from ihBTC2 cells compared to the untreated control group. (P<0.01). F, Phosphorylated Akt and Bcl-2 protein expression of ihBTC2 cells increased significantly after radiation (P<0.05). The up-regulation of these proteins in ihBTC2 cells induced by radiation was inhibited by GSI (P<0.05).

Three hours after 4 Gy irradiation, the Notch1 mRNA from SU3, SU3-5R and ihBTC2 cells increased 1.13±0.12 times (P>0.05), 2.74±0.37 times (P<0.05) and 2.30±0.16 times (P<0.01), respectively, compared to before irradiation (Fig 3A). The Notch ligand Jag1 mRNA from SU3, SU3-5R and ihBTC2 cells increased 1.54±0.41 times (P>0.05), 3.35±0.95 times (P<0.05) and 5.56±0.86 times (P<0.01), respectively, compared to before irradiation (Fig 3B). Forty-eight hours after 4 Gy radiation, the expression of the Notch1 and NICD protein of SU3-5R and ihBTC2 cells increased significantly compared to before radiation (P<0.05; Fig 3C and 3D). These results show that radiation exposure activates Notch1 in the ihBTC2 cell line. Then, the expression of Notch target genes in ihBTC2 cells, including Hes1, Hes2, and Hes4, were detected after radiation exposure. After irradiation, the expression of Hes1 mRNA in ihBTC2 cells was 3.31±0.19 times higher than before. GSI significantly inhibited the up-regulation of Hes1 mRNA from ihBTC2 cells compared to the untreated control group (Fig 3E). These results indicate that Notch1 activation leads to the up-regulation of Hes1, which was abolished by GSI. Phosphorylated Akt and Bcl-2 protein expression of the ihBTC2-radiation group was significantly increased compared with the control group. After inhibition of notch signaling, radiation failed to increase the expression of the p-Akt and Bcl-2 proteins (P<0.05; Fig 3F). These results demonstrate that radiation activates the Notch1/Hes1 signaling pathway in ihBTC2 cells. Then, it upregulates the downstream protein pAkt and anti-apoptotic protein Bcl-2, which plays an anti-apoptotic role.

### The radiosensitivity of ihBTC2 cells is improved by blocking the Notch signal pathway

**Fig 4:**
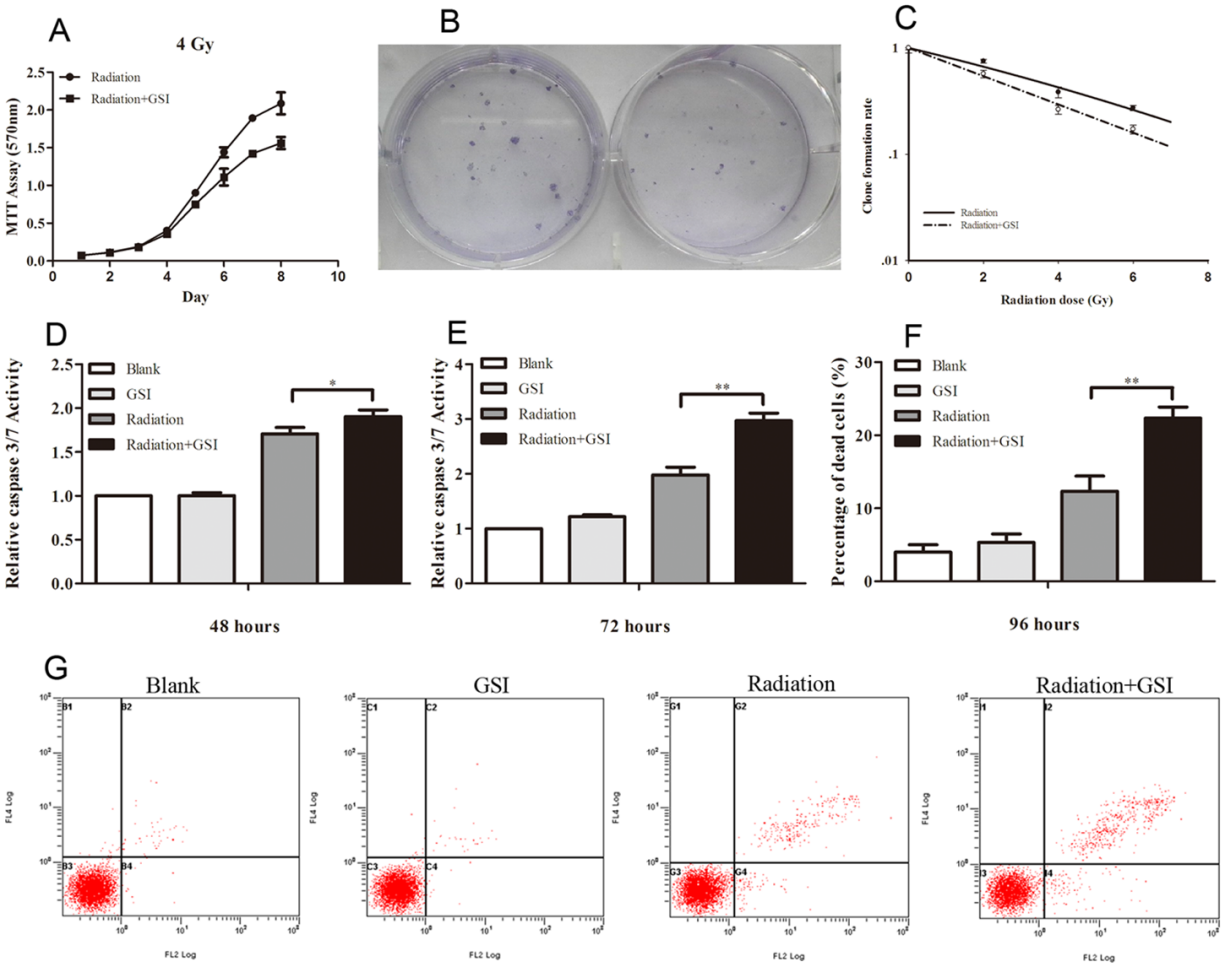
Radiosensitivity of ihBTC2 is improved by inhibiting the Notch signaling pathway. A-B, The cell proliferation (A) and the colony forming ability (B) of the radiation combined with GSI group is decreased compared to the radiation group (P<0.05). C, The SF2 of the untreated and GSI-treated ihBTC2 cells were 0.57±0.05 and 0.75±0.03 (P<0.05), respectively. D-E, Compared to the radiation group, the caspase-3/7 activity of the radiation combined with GSI group was significantly increased after 48 hours (D) and 72 hours (E). F, Trypan blue staining indicated that the dead cell rate of the radiation combined with GSI group was significantly higher compared to the radiation group after 96 hours (P<0.05). G, After 24 hours, the Annexin V staining of the blank, GSI, radiation and radiation combined with GSI group were 0.22%±0.04%, 0.29%±0.04%, 4.60±0.43% and 11.68±0.52%, respectively.

The cell proliferation curve of ihBTC2 cells showed that the proliferation rate of the radiation combined with the GSI group became gradually lower than the radiation group on the fifth day. On the eighth day, GSI severely impaired the growth of ihBTC2 cells exposed to radiation (P<0.05; Fig 4A). The cell radiation dose survival curve indicated that the SF2 of untreated and GSI-treated ihBTC2 cells were 0.57±0.05 and 0.75±0.03 (P<0.05), respectively (Fig 4B and 4C). The SER of GSI was 1.47. The caspase-3/7 activation of ihBTC2 cells was not improved using GSI alone. However, increased caspase-3/7 activity was seen in the radiation combined with the GSI group at 48 and 72 hours after radiation (Fig 4D and 4E; P<0.05, P<0.01). Trypan blue staining analysis demonstrated that 96 hours after radiation, the dead cell rates of the radiation group and the radiation combined with GSI group were 12.3±2.1% and 22.3±1.5%, respectively (P<0.01; Fig 4F). GSI promoted the apoptosis of ihBTC2 cells after radiation. Twenty-four hours after radiation, the Annexin V activity of the radiation group and the radiation combined with GSI group were 4.60±0.43% and 11.68±0.52%, respectively (P<0.01). This result indicates that GSI effectively promoted the early apoptosis of ihBTC2 cells after radiation (Fig 4G).

## Discussion

The relationship between normal host tissue in the tumor microenvironment and tumor radiation resistance has become an important issue in cancer research. Previous studies focused on tumor-associated macrophages (TAMs) and tumor-associated fibroblasts (TAFs). Mounting evidence implicating radiotherapy-induced macrophage aggregation has been paralleled by decreased radiocurability. TAMs in the postirradiated tumor microenvironment express higher levels of Arg-1, COX-2, and iNOS and promote early tumor growth in vivo [10]. Depletion of TAMs by systemic or local injection of the macrophage-depleting liposomal clodronate before radiotherapy increases the antitumor effects of radiation [11]. Furthermore, the reduction of myeloid cell recruitment also enhances the tumor response to radiation [12]. For TAFs, pancreatic stellate cells increase the radiation resistance of pancreatic cancer cells via β1-integrin signaling [13]. After exposure to radiation, TAFs enhance the growth of malignant lung epithelial cells [14]. The radiation resistance of glioma stromal cells (ihBTC2) discussed here is different from previous studies because ihBTC2 cells are a malignant host cell line induced by the tumor stem/progenitor cells SU3. Although they are all host-derived stromal cells in tumors, previous studies only showed that TAMs and TAFs protect tumor cells from radiation, and their own fate remains unknown. However, because ihBTC2 cells were more radiation-resistant than SU3 cells, they should preferentially survive. The majority of gliomas possess inherent resistance mechanisms against radiotherapy and chemotherapy. Similar to drug resistance, radiation resistance must also be overcome. It was previously hypothesized that the high heterogeneity of tumor cells caused the radiation resistance of tumors. In the 21st century, tumor stem cells were regarded as the root of radiation resistance [15]. Currently, the tumor microenvironment theory has garnered the attention of oncologists, and the roles of TAMs and TAFs in protecting tumor cells from radiation has become more attractive. Our finding that malignant host stromal cells in tumors induced by tumor stem/progenitor cells are more radiation resistant than tumor stem cells is a novel finding.

For the malignant transformation of tumor stromal cells, David M. reported the malignant potential of murine stromal cells after the transplantation of different human tumors into nude mice in 1981 [16]. When we transplanted the human glioma stem/progenitor cell line SU3 into the brain, abdomen, subcutaneous tissue and liver of nude mice, we had established the malignant host cell lines ihBTC; ihCTC, derived from abdominal macrophages; ihSTC, derived from hypodermic fibroblasts; and ihHTC, derived from hepatic dendritic cells, respectively [17]. These results suggest that this malignant transformation was not restricted by the transplant sites. Notably, the mouse macrophage-like cell line, RAW264.7, which is widely used in tumor research [18, 19], formed tumors in all transplanted nude mice (10/10) in our previous study [17]. Some studies only focused on the behaviors of tumor cells and did not determine the fate of TAMs and TAFs when exploring their roles in promoting tumor growth or radiation protection. TAMs and TAFs are immortalized host cells induced by primary tumor cells, which may also possess tumorigenesis potential. Although the above results were obtained from an animal model and not clinical patients, the literature from recent decades reported the presence of donor cell-derived tumors in allogeneic hematopoietic stem cell transplants and bone marrow transplant recipients [20, 21]. Although the specific mechanism may not be identical, these donor-derived tumor cells were derived from normal cells in the microenvironment. Therefore, regarding research into tumor cell resistance to radiation, it is not sufficient to only study cancer cells, including tumor stem cells. According to our data, even if the glioma stem/progenitor cell line SU3, which is a tumor initiating cell line, is completely eliminated by radiation, ihBTC2 cells can survive, due to their greater resistance to radiation compared to SU3 cells.

Because malignant ihBTC2 cells are induced by glioma stem/progenitor cells, research into the mechanisms of radiation resistance should start with the signaling pathways of glioma stem cells. The Notch pathway is an important signaling pathway in glioma stem cells. Seventeen genes represent active Notch signaling components, including NOTCH1, NOTCH3, HES1, MAML1, DLL-3, and JAG2, which are enriched in the proneural subtype of glioma stem cells [22]. Notch pathway inhibition depletes CD133+ glioma stem cells through reduced proliferation and increased apoptosis associated with decreased AKT and STAT3 phosphorylation [23]. Wang reported that activated Notch up-regulated the anti-apoptotic protein activity of phosphorylated Akt and Mcl-1 and promoted the radiation resistance of glioma stem cells [24]. Our present study verified that the Notch1 mRNA from ihBTC2 cells increased significantly after radiation. Further research showed that activation of the Notch1/Hes1 pathway up-regulated the anti-apoptotic proteins phosphorylated Akt and Bcl-2 in ihBTC2 cells after radiation and resulted in inBTC2 radioresistance. In addition, inhibition of the Notch pathway with GSI promotes the apoptosis of ihBTC2 cells after radiation and renders ihBTC2 cells more sensitive to radiation at clinically relevant doses.

In summary, our data indicate that the malignant host cell line ihBTC2 in the tumor microenvironment induced by glioma stem/progenitor cells is more resistant to radiation than the glioma stem/progenitor cell line SU3. The radiation resistance of the malignant host cell line ihBTC2 was regulated by the Notch pathway. Gamma secretase inhibitors improved the sensitivity to radiation by inhibiting the Notch pathway. These results improve our understanding of the mechanisms of radiation resistance of gliomas and are a basis for the development of more effective strategies for radiotherapy.
